# Targeted DNA methylation in pericentromeres with genome editing-based artificial DNA methyltransferase

**DOI:** 10.1371/journal.pone.0177764

**Published:** 2017-05-18

**Authors:** Taiga Yamazaki, Yu Hatano, Tetsuya Handa, Sakiko Kato, Kensuke Hoida, Rui Yamamura, Takashi Fukuyama, Takayuki Uematsu, Noritada Kobayashi, Hiroshi Kimura, Kazuo Yamagata

**Affiliations:** 1 Division of Biomedical Research, Kitasato University Medical Center, Kitasato University, Kitamoto, Saitama, Japan; 2 Faculty of Biology-Oriented Science and Technology, KINDAI University, Kinokawa, Wakayama, Japan; 3 Cell Biology Unit, Institute of Innovative Research, Tokyo Institute of Technology, Midori-ku, Yokohama, Japan; Saint Jude Children's Research Hospital, UNITED STATES

## Abstract

To study the impact of epigenetic changes on biological functions, the ability to manipulate the epigenetic status of certain genomic regions artificially could be an indispensable technology. “Epigenome editing” techniques have gradually emerged that apply TALE or CRISPR/Cas9 technologies with various effector domains isolated from epigenetic code writers or erasers such as DNA methyltransferase, 5-methylcytosine oxidase, and histone modification enzymes. Here we demonstrate that a TALE recognizing a major satellite, consisting of a repeated sequence in pericentromeres, could be fused with the bacterial CpG methyltransferase, SssI. ChIP-qPCR assays demonstrated that the fusion protein TALMaj-SssI preferentially bound to major chromosomal satellites in cultured cell lines. Then, TALMaj-SssI was expressed in fertilized mouse oocytes with hypomethylated major satellites (10–20% CpG islands). Bisulfite sequencing revealed that the DNA methylation status was increased specifically in major satellites (50–60%), but not in minor satellites or other repeat elements, such as Intracisternal A-particle (IAP) or long interspersed nuclear elements-1 (Line1) when the expression level of TALMaj-SssI is optimized in the cell. At a microscopic level, distal ends of chromosomes at the first mitotic stage were dramatically highlighted by the mCherry-tagged methyl CpG binding domain of human MBD1 (mCherry-MBD-NLS). Moreover, targeted DNA methylation to major satellites did not interfere with kinetochore function during early embryonic cleavages. Co-injection of dCas9 fused with SssI and guide RNA (gRNA) recognizing major satellite sequences enabled increment of the DNA methylation in the satellites, but a few off-target effects were also observed in minor satellites and retrotransposons. Although CRISPR can be applied instead of the TALE system, technical improvements to reduce off-target effects are required. We have demonstrated a new method of introducing DNA methylation without the need of other binding partners using the CpG methyltransferase, SssI.

## Introduction

Methylated cytosines in CpG dinucleotides (5mC) play crucial roles in various biological phenomena through regulating gene expression, and aberrant DNA methylation leads to diseases and developmental defects [1]. For instance, hypermethylation of CpG islands located in tumor repressor gene promoters and hypomethylation of satellite DNA and retrotransposons are unique characters frequently observed in cancerous cells [2]. Also, mice carrying mutations in a DNA methyltransferase (*Dnmt*) gene reveal embryonic lethality [3, 4]. As illustrated by Waddington, cellular differentiation and development is an irreversible process [5] and thought to be controlled by some epigenetic mechanism. It is now well accepted that dynamic changes in DNA methylation occur during stem cell differentiation, preimplantation development, and primordial germ cell (PGC) development [6–8]. Once these cells are differentiated or developed, cellular or tissue-specific DNA methylation is also established [9]. For instance, we previously found that DNA methylation in centromeres (minor satellites) and pericentromeres (major satellites) is hypomethylated (10–20% CpG) specifically in germ cells, including sperm, oocytes, and preimplantation embryos, whereas all somatic cells analyzed had highly methylated DNA [10]. To study the impact of DNA methylation changes in certain genomic regions of interest, such as cancer-related genes or centromeres and pericentromeres, a methodology to introduce DNA methylation specifically is needed.

Currently, there are reports about upregulating DNA methylation. Mouse or human Dnmt3a fused with a zinc-finger protein that recognizes specific DNA sequences enables the upregulation of DNA methylation in the *IE175* gene promoter region derived from the Herpes simplex virus [11], *VEGF-A* promoter [12], and *SOX2* promoter [13, 14] in cultured cells. Also, synthetic molecule composed of chromatin binding domain of SUV39H1 and JMJD2D enabled to modify histone H3K9me3 marks of heterochromatin [15]. Recently, TALE and CRISPR technology have been widely applied not only in editing genomes, but also in regulating transcription activity [16–18] and vital labeling of specific genome loci [19, 20]. Several studies have used these technologies to edit epigenomes. TALE-TET1 demethylates *RHOXF2*, *HBB* [21] and TALE-DNMT3a-3L enable the induction of DNA methylation in the *p16* (*CDKN2A*) gene [22]. Optogenetic approach to modifying DNA methylation of Ascl1 promoter were also reported [23]. With the CRISPR system, the dCas9-p300 core domain fusion protein upregulates the gene expression of *IL1RN*, *MYOD*, and *OCT4* via the acetylation of H3K27 that locates upstream of target genes [24], and dCas9-DNMT3A upregulates DNA methylation of the *IL6ST* and *BACH2* promoters [25]. A recent study also revealed that dCas9-Tet1 or dCas9-Dnmt3a enables the editing of targeted CpG methylation [26].

It would be reasonable to introduce DNA methylation in mammalian cells by *de novo* type CpG methyltransferase DNMT3; however, DNMT3 requires DNMT3L binding for the efficient induction of DNA methylation [27–29]. SssI is a bacterial CpG methyltransferase that catalyzes the transfer of methyl groups to the cytosine of CpG dinucleotides. We attempted to take advantage of the *SssI* gene to upregulate hypomethylated regions. Here we focused on upregulating DNA methylation in mouse pericentromere major satellite sequences. TALE recognizing 15 nucleotides of a major satellite, originally developed by Miyanari et al. [30], was fused with SssI, and then its ability to induce DNA methylation in a major satellite was assessed.

## Materials and methods

### Cell culture

ES cell lacking Dnmt1, Dnmt3a and Dnmt3b (Dnmt TKO ES cell, AES0146) [31] was obtained from RIKEN BRC and cultured in GMEM (Wako Pure Chemical Industries, Ltd., Japan) supplemented with 15% FBS, 0.1mM NEAA (Wako Pure Chemical Industries, Ltd., Japan), 1mM Sodium pyruvate (Wako Pure Chemical Industries, Ltd., Japan), LIF (Wako Pure Chemical Industries, Ltd., Japan) and 0.1mM 2-mercaptoethanol (Wako Pure Chemical Industries, Ltd., Japan) on gelatin coated dish. C3H10T1/2 (JCRB0003) was obtained from the JCRB cell bank (National Institute of Biomedical Innovation, Health and Nutrition, Japan) and cultured in DMEM (Thermo Fisher Scientific Inc., MA, USA) supplemented with 10% fetal bovine serum, 100 U/mL penicillin, and 0.1 mg/mL streptomycin. These cells were cultured at 37°C under 5% CO_2_ in air.

### Plasmid construction and transfection

Plasmids encoding major satellite recognition TALE [30], dCas9 [19], and guide RNA (gRNA) encoding vector pgRNA-humanized [19], were purchased from Addgene (plasmid # 47878, 44246, and 44248, respectively). TALMaj and dCas9 DNA fragments were amplified by PCR and ligated into pcDNA3.1 poly (A) vector [32], pTetOne vector (Takara Bio Inc., Japan) or PiggyBac cDNA expression vector (System Biosciences, Inc., CA, USA) together with a DNA fragment encoding SssI CpG methyltransferase. In 2007, we successfully cloned the *SssI* gene, which has four amber mutations in its open reading frame, and fixed these amber mutations into normal sense codons [33]. An enzymatically inactive SssI, T313D mutant, was prepared using site-direct mutagenesis by replacing the threonine located at position 313 of SssI with aspartic acid [34]. Enzymatically active (WT) and inactive (T313D) SssI were tagged with FLAG-HA or 3× FLAG at the C-terminus. DNA sequences encoding a gRNA expression unit containing the T7 promoter, *Bbs*I digestion site, dCas9 binding hairpin, including A-T flip and stem extension combined [19], and *Streptococcus pyogenes* terminator, was synthesized (Thermo Fisher Scientific Inc., MA, USA) and inserted between the *BstX*I and *Xho*I sites of a pgRNA-humanized vector. A DNA fragment encoding major satellite targeting gRNA was synthesized with oligonucleotides [20]. Two nucleotide fragments were annealed and ligated into the *Bbs*I digested gRNA expression plasmid. A plasmid encoding mCherry-MBD-NLS, Histone H2B-EGFP and EGFP-CENPC were prepared previously [35]. Dnmt TKO ES cell line stably expressing both mCherry-MBD-NLS and TALMaj-SssI were obtained by introduction of PiggyBac cDNA expression vector and Super PiggyBac Transposase Expression vector (System Biosciences, Inc., CA, USA) with Lipofectamin3000. Antibiotic-resistant clones were selected and used in the study. TALMaj-SssI encoding pTetOne was transfected with Lipofectamin3000 (Thermo Fisher Scientific Inc., MA, USA) into Dnmt TKO ES cell and C3H10T1/2 cell. Doxycycline was added to transfected cells at 1 μg/mL concentration to induce TALMaj-SssI expression.

### ChIP-qPCR

As we could not establish stable ES cell lines expressing TALMaj-SssI WT (for unknown reasons), we used ES cells with TALMaj-SssI (T313D) for the ChIP-qPCR analysis. Dnmt TKO ES cells expressing both mCherry-MBD-NLS and TALMaj-SssI (T313D) 3×FLAG were cross-linked in 1% formaldehyde containing DMEM for 5 min and washed with NP40 buffer containing 10 mM Tris-HCl (pH 8.0), 10 mM NaCl, and 0.5% NP-40. Fixed cells were lysed with SDS buffer containing 50 mM Tris-HCl (pH 8.0), 1% SDS and 10 mM EDTA, then suspended in ChIP dilution buffer containing 50 mM Tris-HCl (pH 8.0), 167 mM NaCl, 1.1% Triton X-100 and 0.11% sodium deoxycholate. Samples were sonicated for 5 min and centrifuged at 20,000 *g* for 10 min. The supernatant was incubated with anti-FLAG antibody (F1804, Sigma-Aldrich Co. LLC., St. Louis, MO, USA) prebound to Dynabeads M280 Sheep anti-Mouse IgG (Thermo Fisher Scientific Inc., Waltham, MA, USA) at 4°C overnight with rotation. The immune complexes were washed twice with low-salt RIPA buffer containing 150 mM NaCl and high-salt RIPA buffer containing 500 mM NaCl, then washed twice with TE buffer. DNA elution was performed with ChIP elution buffer containing 10 mM Tris-HCl (pH 8.0), 300 mM NaCl, 5 mM EDTA, and 0.5% SDS. After incubation at 65°C to reverse the cross-links, RNase A and proteinase K were added to precipitate the DNA. DNA was recovered by phenol–chloroform and ethanol precipitation. DNA samples were analyzed by qPCR with the following sets of primers as previously described [30, 36]:

5′-GATTTCGTCATTTTTCAAGTCGTC -3′ and

5′-TTTAGAAATGTCCACTGTAGG-3′ for the major satellite;

5′-ACTCATCTAATATGTTCTACAGTG -3′ and

5′-AAAACACATTCGTTGGAAACGGG-3′ for the minor satellite;

5′-ACACACCAGAAGAGGGCATC -3′ and

5′-GAGCACCTGACTGCTCTTCC-3′ for the SineB2;

5′-AACCTACTTGGTCAGGATGGATG-3′ and

5′-AGTGCAGAGTTCTATCAGACCTTC-3′ for the Line1;

5′-CCCCGTCCCTTTTTTAGGAG-3′ and

5′-CTCCATGTGCTCTGCCTTCC-3′ for the IAP.

### Microinjection of *in vitro* transcribed RNA into embryos

The gRNA expression plasmid was linearized by *Xho*I digestion, and gRNA was synthesized with a RiboMAX Large Scale RNA Production System-T7 (Promega Co., WI, USA). RNA encoding TALMaj-SssI (WT or T313D), dCas9-SssI (WT or T313D), mCherry-MBD-NLS, and Histone H2B-EGFP were synthesized with an *in vitro* transcription kit using linearized plasmids, as described previously [32, 33, 37]. Cumulus-intact Metaphase-II arrested oocytes were obtained from 8–12-week-old ICR mice and inseminated with capacitated sperm (50 cells/μL) in TYH medium and incubated for 60 min. Fertilized oocytes developed to Anaphase-II or Telophase-II stage were recovered and injected with RNA, and then incubated at 37°C under 5% CO_2_ in air. We expressed TALMaj-SssI at three different levels of expression (low, mid, and high) by injecting various concentrations (2 ng, 10 ng, and 50 ng/μL) of RNA encoding TALMaj-SssI. We also set three different levels of dCas9-SssI expression by injecting 12 ng, 60 ng, and 300 ng/μL of RNA mixed with a same concentration of major satellite targeting gRNA (12 ng, 60 ng, and 300 ng/μL). For imaging global levels of DNA methylation and chromatin, we injected RNA encoding mCherry-MBD-NLS (5 ng/μL) and Histone H2B-EGFP (10 ng/μL)　with effector (TALMaj or dCas9)-SssI encoding RNA. This study was carried out in strict accordance with the recommendations in the Guide for the Care and Use of Laboratory Animals of the KINDAI University. The protocol was approved by the Committee on the Ethics of Animal Experiments of the KINDAI University (Permit Number: KABT-27-004). All mice were euthanized by cervical dislocation, and all efforts were made to minimize suffering.

### Immunostaining

Cells were cultured in coverglass chambers (AGC Techno Glass Co., Ltd, Japan) treated with Cellmatrix Type I-P collagen (Kurabo Industries Ltd, Japan) or fibronectin (Wako Pure Chemical Industries, Ltd., Japan), Poly-L-ornithine (Sigma-Aldrich Co. LLC., MO, USA)) and laminin (Wako Pure Chemical Industries, Ltd., Japan). Cultured cells were fixed with 4% paraformaldehyde for 30 min and permeabilized with PBS containing 0.1% TritonX-100 for 15 min. After blocking with PBS containing both 1% bovine serum albumin and 0.1% Tween 20 for 30 min, cells were treated with anti-FLAG antibody (F1804, Sigma-Aldrich Co. LLC., MO, USA) for 1 h, and then incubated with Alexa Fluor 555 conjugated anti-mouse IgG (H+L), F (ab′) 2 fragment (#4409, Cell Signaling Technology, Inc., MA, USA) for 1 h. Cells were counterstained with DAPI (1 μg/mL) and observed with imaging system composed of an inverted microscope equipped with a CSU-W1 Nipkow disk confocal scanning unit (Yokogawa Electric Co., Japan) or a fluorescent microscope (FSX100, Olympus Co., Japan).

### Live-cell imaging

Fertilized oocytes expressing mCherry-MBD-NLS, Histone H2B-EGFP, and effector (TALMaj or dCas9)-SssI were placed on an imaging system composed of an inverted microscope equipped with a CSU-W1 Nipkow disk confocal scanning unit (Yokogawa Electric Co., Japan) at 37°C with 5% CO_2_ in air. Images were captured as vertical sections (at approximately 1–5 μm intervals) using an EMCCD camera (iXON3 DU897E-CS0-#BV-Y, Andor Technology Ltd, UK) and Z-axis motor. A silicon oil-immersion objective lens (30×, 60×, or 100×; Olympus Co., Japan) was used for live-cell imaging.

### Quantification of imaging data

Imaging data (Z-axis 3D images) obtained from time-lapse analysis were stacked into 2D images for each time-point using MetaMorph software (Molecular Devices LLC., CA, USA). Data were analyzed using MetaMorph software or ImageJ software (http://imagej.nih.gov/ij). To construct the 3D images, we used Volocity software (PerkinElmer, MA, USA). To acquire the DNA methylation data from the first mitosis movie, multiple embryo images were assembled into one movie with the same dynamic range of fluorescence intensity. The intensity of mCherry-MBD-NLS in mitotic chromosomes was measured in the regions of interest (ROIs). The shapes of the chromosomes in the images were extracted using the threshold setting, and ROIs were placed over them manually. The mean fluorescence intensity was then measured at all times for each mitotic stage. To acquire the heterochromatin index from the 2-cell nucleus images, each round shape of a nucleus was defined as an ROI and extracted using the threshold setting, and then the fluorescent signal of the ROI was measured manually. The heterochromatin index [35] was calculated using the following formula: Heterochromatin index = standard deviation (SD) of the signal intensity / average signal intensity. For detecting abnormal chromosome segregation (ACS), the numbers of embryos showing misaligned chromosomes or lagging chromosomes labeled with H2B-EGFP were counted during the 1-cell to 8-cell stages [38]. Results were obtained from multiple experiments. For statistical analysis, we used GraphPad Prism version 6.02 (GraphPad Software, CA, USA). Two-way ANOVA, the Mann–Whitney *U* test or chi-square test was used for statistical analysis up to the composition of data sets; *P*<0.05 was considered to be significant.

### Bisulfite sequencing

Embryos injected with RNA encoding effector (TALMaj or dCas9)-SssI, mCherry-MBD-NLS, and Histone H2B-EGFP were cultured for 72 h, and embryos developed to the morula/blast stage were recovered for a bisulfite reaction performed with an EZ DNA Methylation-Direct Kit (Zymo Research Co., CA, USA) according to the manufacturer’s instructions. Briefly, cells were placed directly into PCR tubes containing lysis buffer and incubated at 50°C for 20 min, and then subjected to a bisulfite reaction. After bisulfite treatment, eluted DNA was amplified by PCR using the following sets of primers:

5′-GGAATATGGTAAGAAAATTGAAAATTATGG-3′ and

5′-CCATATTCCAAATCCTTCAATATACATTTC-3′ for the major satellite;

5′-TAGAATATATTAGATGAGTGAGTTATATTG-3′ and

5′-ATTATAACTCATTAATATACACTATTCTAC-3′ for the minor satellite;

5′-TTGATAGTTGTGTTTTAAGTGGTAAATAAA-3′ and

5′-AAAACACCACAAACCAAAATCTTCTAC-3′ for the IAP;

5′-TAGGAAATTAGTTTGAATAGGTGAGAGGT-3′ and

5′-TCAAACACTATATTACTTTAACAATTCCCA-3′ for the Line1.

The PCR reaction for amplifying major and minor satellites was 40 cycles of 95°C for 30 s, 60°C for 60 s, and 68°C for 20 s. PCR conditions for amplifying IAP and the Line1 fragment were 35 cycles of 95°C for 60 s, 60°C for 60 s, 72°C for 60 s, followed by 72°C for 3 min. The amplified DNA fragments were subcloned into a pGEM-T Easy vector (Promega Co., WI, USA), and 10–20 clones derived from each group of bisulfite-treated genomic DNA were sequenced. Methylation sites were visualized using the web-based DNA methylation analysis tool “QUMA” (http://quma.cdb.riken.jp/) [39]. Statistical significance was quantified using the Mann–Whitney *U* test performed using QUMA.

## Results

### Design and validation of major satellite-targeted DNA methyltransferase SssI, TALMaj-SssI

**Fig 1:**
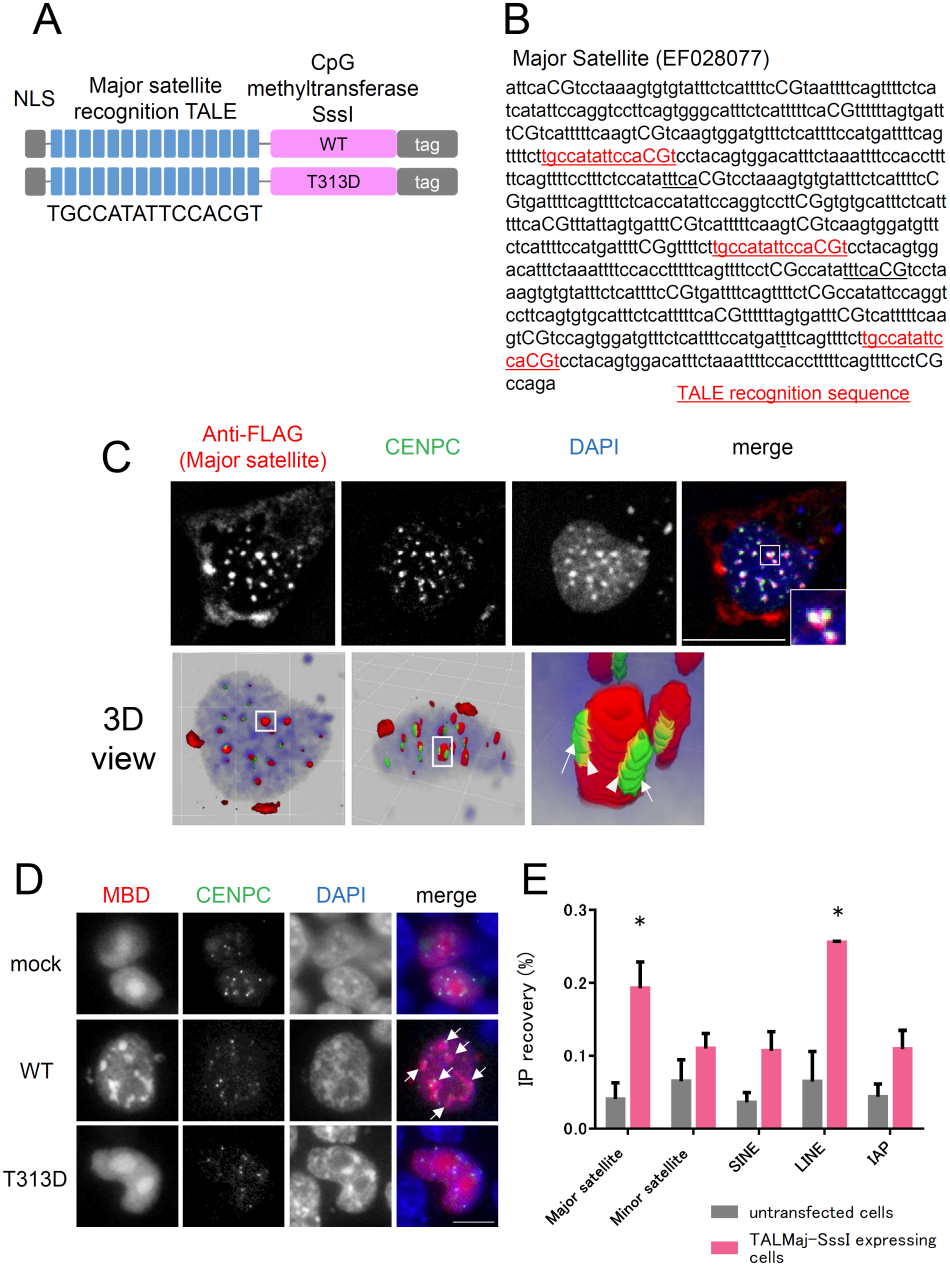
Confirmation of target-preferential CpG methylation activity of TALMaj-SssI. (A) Structure of major satellite recognition TALE and bacterial CpG methyltransferase, SssI, fusion protein. Major satellite recognition TALE, originally designed by Miyanari et al., were fused with 3×FLAG or FLAG-HA that was tagged enzymatically active (WT) or inactive (T313D) SssI DNA methyltransferase. (B) TALE recognition sequence of the major satellite repeat. Red character indicated TALE recognition 15 nucleotides in the major satellite sequences (GenBank: EF028077). (C) TALMaj-SssI localization at pericentromere. Immunostaining of FLAG-tagged TALMaj-SssI (WT) expressing mouse C3H10T1/2 cultured cells. Cells were transfected TALMaj-SssI-3×FLAG expressing plasmid together with EGFP-CENPC-expressing plasmid. Cells were fixed 48h after transfection and then stained with anti-FLAG antibody. Enlarged images of a single heterochromatin focus in nucleus are shown in the inset in the upper panel. The bottom panel indicates 3D reconstitution images of the staining data. The single heterochromatin focus within the square is enlarged in the right panel. Arrows indicate CENPC which locates around FLAG signal. Arrowheads indicate CENPC which shows slight overlap with FLAG signals. Red: FLAG, Green: EGFP-CENPC, Blue: DAPI. Scale bar represents 20μm. (D) Upregulation of pericentromeric DNA methylation by TALMaj-SssI expression. TALMaj-SssI (mock vector, WT, or T313D), mCherry-MBD-NLS, and EGFP-CENPC were introduced into Dnmt TKO ES cells. DNA methylation, kinetochores, and DNA are shown by mCherry-MBD-NLS (red), EGFP-CENPC (green), and DAPI (blue), respectively. Arrows indicate induced DNA methylation in DAPI-dense heterochromatin regions. Scale bar represents 20 μm. (E) ChIP-qPCR analysis of TALMaj-SssI-stably expressing Dnmt TKO ES cells. Localization of TALMaj-SssI (T313D)-3×FLAG was analyzed by ChIP-qPCR with anti-FLAG antibody. Untransfected ES cells were used as the control. Asterisks indicate significant differences by two-way ANOVA (*p*<0.05).

We designed fusion proteins composed of a TALE recognizing major satellites [30] and enzymatically active (WT) or inactive (T313D) SssI CpG methyltransferase (Fig 1A and 1B). We first confirmed the localization of TALMaj-SssI in cultured cells. Plasmids encoding 3×FLAG-tagged TALMaj-SssI and EGFP-tagged CENPC were introduced into C3H10T1/2 cells. TALMaj-SssI was immunostained with anti-FLAG antibody, and 3D images were reconstructed. TALMaj-SssI was detectable in both the nucleus and cytoplasm. In the nucleus, DAPI-dense signals were well stained with anti-FLAG antibody, and CENPC signals were localized around these heterochromatin foci (Fig 1C). Although there was slight overlap of the TALMaj-SssI and CENPC signals, which indicated the kinetochore region comprising the minor satellite repeat (arrowheads in panel), TALMaj-SssI was enriched mainly in pericentromeres which are DAPI-dense regions comprising major satellite repeats [40].

Next, we introduced TALMaj-SssI into Dnmt (Dnmt1, Dnmt3a, and Dnmt3b) triple knock out ES cell (Dnmt TKO ES cell), which has global hypomethylation including major satellite and minor satellite and identify upregulation of DNA methylation in microscopic level [31] (Fig 1D). To visualize the DNA methylation and centromeric regions, we co-transfected mCherry-MBD-NLS [35] and EGFP-CENPC. Accumulation of MBD signals was observed in DAPI-dense heterochromatin in TALMaj-SssI (WT)-expressing Dnmt TKO ES cells, whereas cells expressing the mutant (T313D) or mock control exhibited a uniform distribution of MBD signals in the nuclei except for the nucleoli. CENPC signals were localized around DAPI-dense heterochromatin. This result suggests that TALMaj-SssI expression upregulated pericentromeric DNA methylation.

We next used ChIP-qPCR analysis to confirm the binding specificity of TALMaj-SssI (Fig 1E). Dnmt TKO ES cells stably expressing TALMaj-SssI (T313D) under the control of the EF1α promoter were established and analyzed. We found that TALMaj-SssI localized both to the major satellite and Line1 retrotransposon, but not to other repeats. This suggests that TALMaj-SssI localizes to the major satellite preferentially, but has some off-target binding under this experimental condition. Although the reason for the off-target binding of TALMaj-SssI is unclear, it is possible that it was caused by abundant expression. Moreover, Line1 has much more copy number compared with other repeat element we tested. These might be reasons why TALMaj-SssI shows off-target binding to Line1.

### Introduction of pericentromere DNA methylation in mouse preimplantation embryos

**Fig 2:**
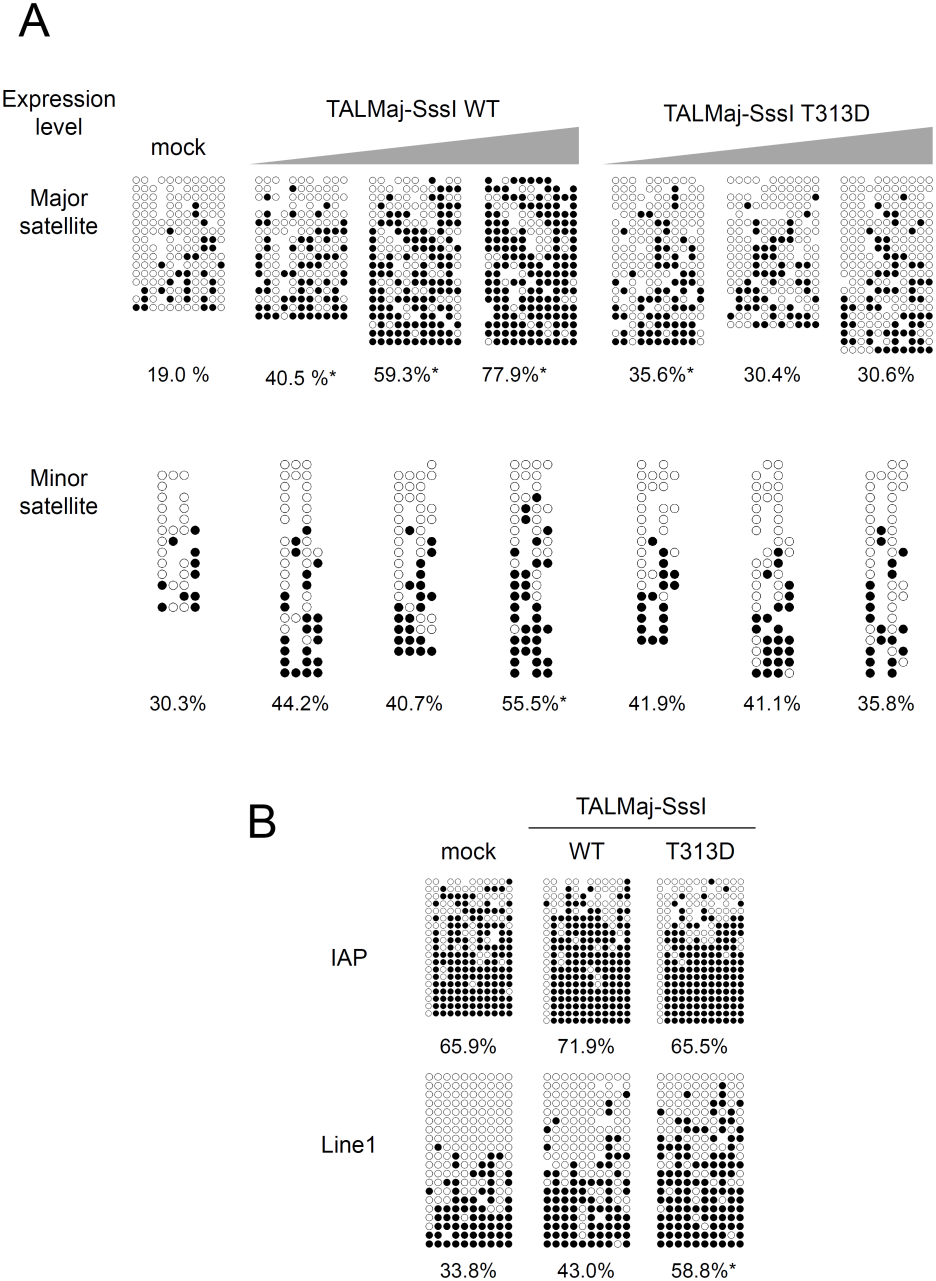
Target dominant upregulation of DNA methylation by TALMaj-SssI expression in mouse embryos. (A) Bisulfite sequencing of TALMaj-SssI-expressing embryos. Methylation of major and minor satellite CpGs was evaluated in TALMaj-SssI embryos. TALMaj-SssI was expressed at 3 levels (low, middle, and high, from left to right) by injecting various concentrations (2, 10, and 50 ng/μL, respectively) of RNAs encoding TALMaj-SssI (WT or T313D) into fertilized embryos. Mock control indicates normal fertilized embryos without injection. Embryos were recovered 3 days after RNA injection. (B) DNA methylation of IAP and Line1 was evaluated in TALMaj-SssI-expressing embryos. Embryos expressing the middle level (10 ng/μL RNA injection) of TALMaj-SssI were evaluated. Mock control indicates normal fertilized embryos without injection. Data are expressed as the percentage of methylated CpG sites relative to all CpG sites. Asterisks indicate significant differences between the mock control and tested group by the Mann–Whitney *U* test.

Because TALMaj-SssI had some off-targets toward the Line1 retrotransposon in our ChIP-qPCR analysis (Fig 1E), we next used bisulfite sequencing to evaluate the DNA methylation of the major and minor satellites under the dose-dependent expression of TALMaj-SssI in mouse preimplantation embryos that were known to have hypomethylated centromeres and pericentromeres based on previous analysis [10]. DNA methylation of the major satellites was upregulated dose dependently in embryos expressing WT TALMaj-SssI, whereas few effects were observed with minor satellite methylation (Fig 2A). We evaluated Line1 and IAP DNA methylation further and found few effects in these sequences (Fig 2B). Therefore, we conclude that TALMaj-SssI upregulates DNA methylation preferentially in the major satellites, but exerts little off-target DNA methylation toward other genomic regions.

**Fig 3:**
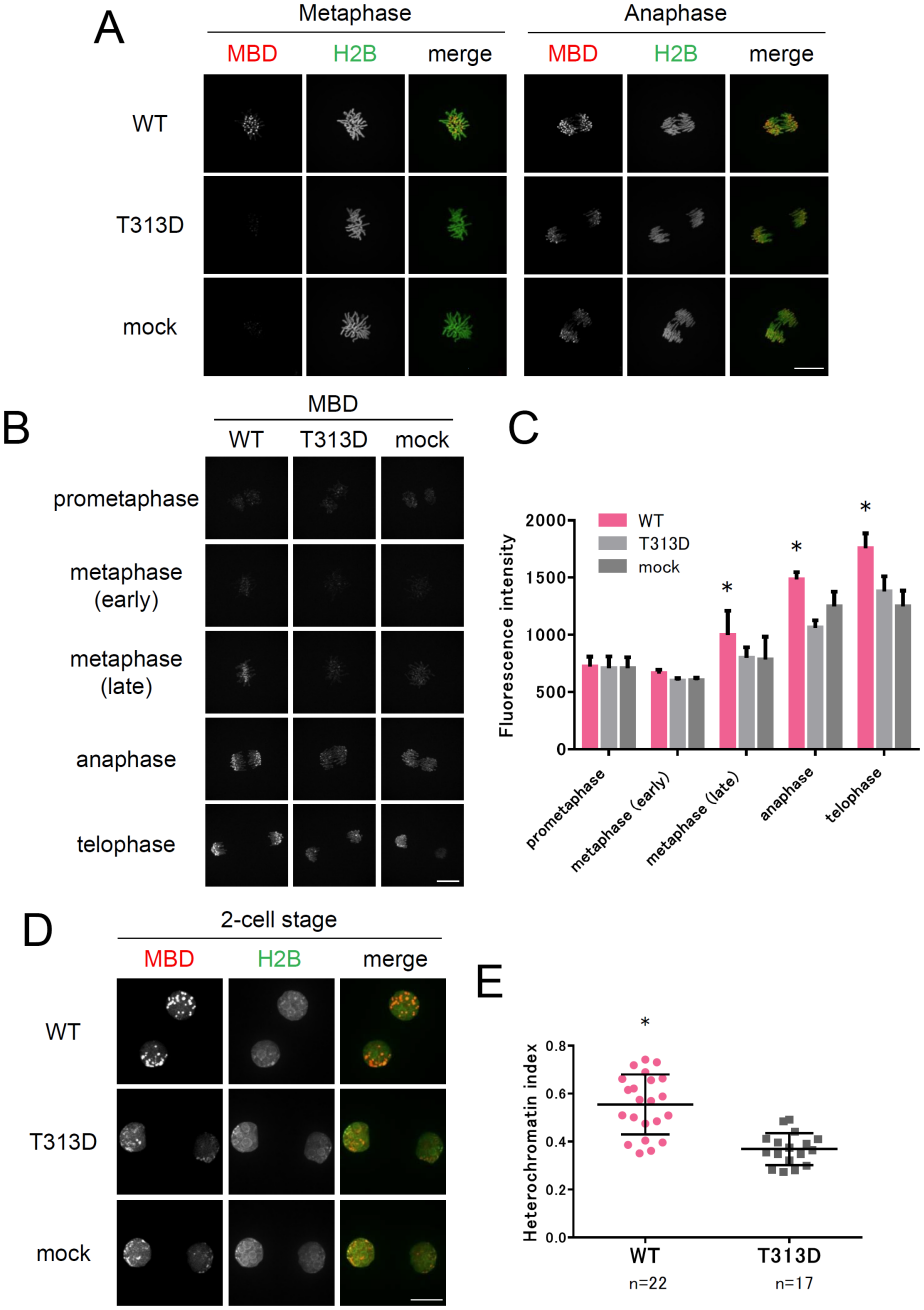
Upregulation of pericentromeric DNA methylation by TALMaj-SssI expression in mouse embryos. (A) Live-cell imaging of DNA methylation in TALMaj-SssI expressing embryos during first mitosis. Fertilized embryos were injected with RNAs encoding TALMaj-SssI, mCherry-MBD-NLS (Red) and H2B-EGFP (Green). Enzymatically active form of CpG methyltransferase SssI (WT) and inactivated SssI (T313D) was tested in this experiment. Mock indicates no injection of TALMaj-SssI. Scale bar represents 20μm. (B) Time-lapse analysis of DNA methylation during the first mitosis. Time-lapse observations were made of embryos expressing both mCherry-MBD-NLS and TALMaj-SssI (WT, T313D, or mock injection). Snapshots indicate DNA methylation (MBD) at each mitotic stage. Scale bar represents 20 μm. (C) Quantification of DNA methylation in embryos expressing TALMaj-SssI. MBD fluorescence of chromosomes was quantified at each mitotic stage. Each data point represents the analysis of 3 embryos. Asterisks indicate significant differences by two-way ANOVA (*p*<0.05). (D) Live-cell imaging of DNA methylation status in TALMaj-SssI expressing 2-cell embryos. Enzymatically active (WT) and inactive (T313D) TALMaj-SssI was expressed in 2-cell embryos with mCherry-MBD-NLS (Red) and H2B-EGFP (Green). 2-cell images were captured about 4–5 h after 2-cell division. Scale bar represents 20μm. (E) Quantification of the heterochromatin index (Ueda et al, 2014) in embryos expressing TALMaj-SssI WT, T313D, and mCherry-MBD-NLS. Fluorescent signal of mCherry-MBD-NLS was evaluated in 2-cell embryos obtained 17 h after 2-cell division. Asterisks indicate significant difference by Mann-Whitney *U* test (*p*<0.05).

We next capture the dynamics of DNA methylation induction, DNA methylation status and patterns in the nucleus were visualized using mCherry-MBD-NLS [35]. RNAs encoding TALMaj-SssI, mCherry-MBD-NLS, and Histone H2B-EGFP were co-injected into mouse fertilized embryos, and the fluorescent signals of mCherry and EGFP were observed during the 1-cell to morula/blast developmental stage. At the first mitotic stage, mCherry-MBD-NLS were strongly accumulated at the distal ends of chromosomes in embryos expressing WT TALMaj-SssI, but not in the embryos injected with the T313D mutant (Fig 3A and 3B and S1 and S2 Movies). Time-lapse analysis revealed that the induction of DNA methylation by TALMaj-SssI occurred mainly in late metaphase stages during the first mitosis (Fig 3B and 3C). It is unclear why this DNA methylation occurs strictly with this specific mitotic stage. In 2-cell nuclei, MBD signals also accumulated at the heterochromatin foci locating on the peripheral part of the nucleus (Fig 3D and S1 Movie). We next calculated the heterochromatin index with the MBD signals. The heterochromatin index is defined as the coefficient of variation of signal patterns in the nucleus [35]. The score fluctuates with fluorescent accumulation in heterochromatin or numbers of heterochromatin foci in the nucleus. Embryos expressing WT TALMaj-SssI indicated a higher heterochromatin index score (Fig 3E). This result suggested that heterochromatin organization is influenced by the upregulation of DNA methylation.

Taken together, these results suggested that DNA methylation was upregulated in pericentromeres of embryos expressing the WT TALMaj-SssI, and that this activity was dependent on the enzymatic activity of SssI. Moreover, this induction of DNA methylation was as drastic as it is able to capture by fluorescent microscope.

**Fig 4:**
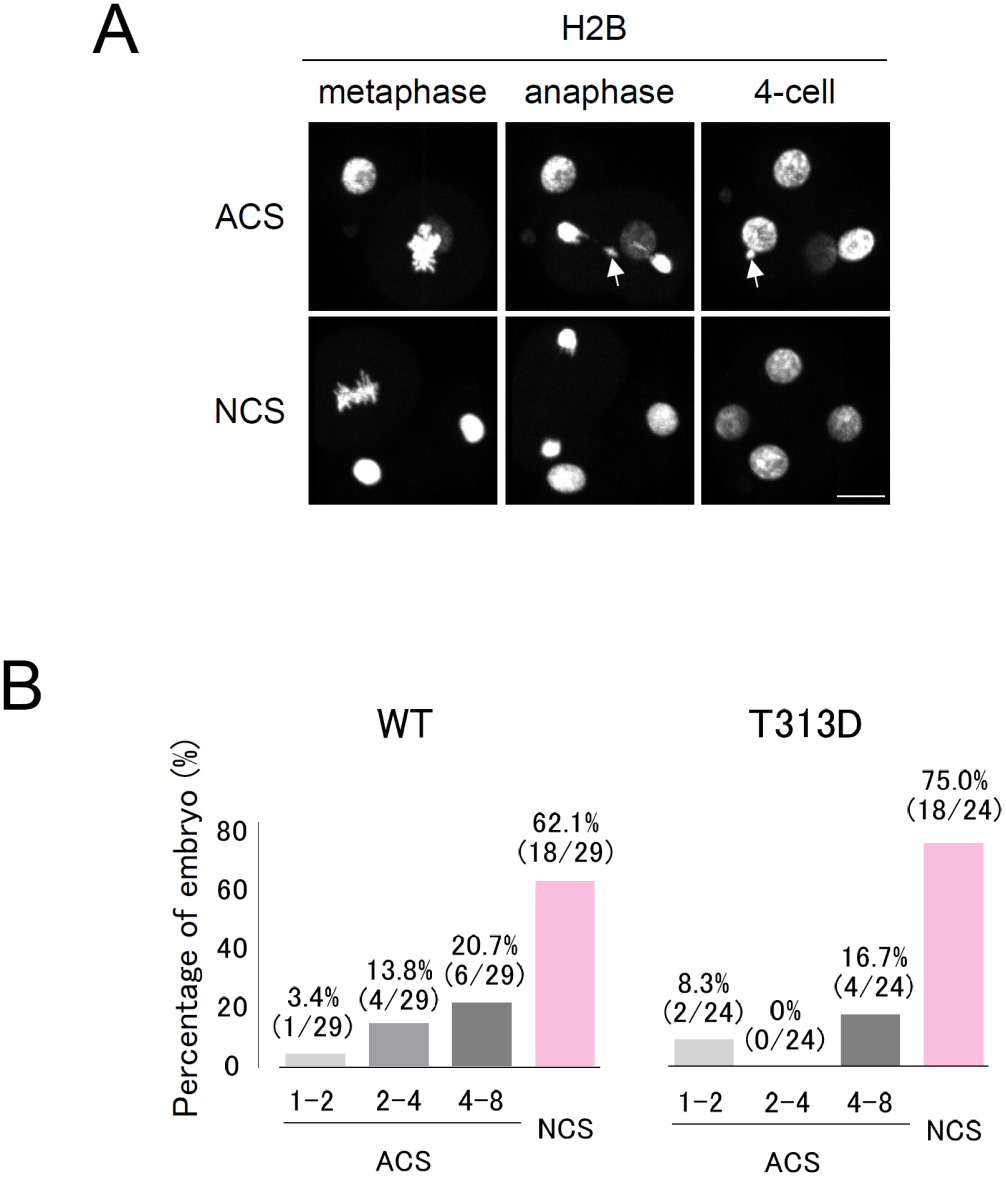
Upregulation of major satellite DNA methylation does not hamper chromosome segregation in preimplantation embryos. (A) Chromosome segregation patterns observed in embryos expressing the TALMaj-SssI (WT) fusion protein. Snapshots of the 2-cell to 4-cell transition are indicated. Embryos were injected with RNAs encoding mCherry-MBD-NLS (5 ng/μL), H2B-EGFP (10 ng/μL) and TALMaj-SssI (WT or T313D; 10 ng/μL) and we observed EGFP fluorescence during the 1-cell to 8-cell stages by live-cell imaging. Embryos were classified into two groups: abnormal chromosome segregation (ACS) and normal chromosome segregation (NCS). Arrows indicate lagging chromosomes. Scale bar = 20 μm. (B) Frequency of ACS in embryos expressing TALMaj-SssI. These were classified as having 1–2 ACS, 2–4 ACS, or 4–8 ACS, based on the timing of ACS at the 1-cell to 2-cell, 2-cell to 4-cell and 4-cell to 8-cell stages, respectively. In all, 29 WT and 24 T313D embryos were assessed for ACS analysis. The ACS frequencies in these three groups were compared with embryos expressing the WT or T313D proteins by chi-squared test. No statistically significant difference was observed.

Although there was leakage of DNA methylation to minor satellites when an excess amount of TALMaj-SssI was expressed, moderate TALMaj-SssI expression ensured target DNA methylation to major satellites without mis/upregulation of DNA methylation in minor satellites (Fig 2A). Furthermore, to evaluate whether TALMaj-SssI expression had some effect on minor satellites or kinetochores under these experimental conditions we analyzed chromosome segregation errors with embryos expressing moderate levels of WT or T313D TALMaj-SssI (Fig 4). Chromosomes labeled with H2B-EGFP were monitored using live-cell imaging from the 1-cell to 8-cell stages and the numbers of embryos with misaligned or lagging chromosomes were counted (Fig 4A). This showed that there were no significant differences in ACS between the embryos expressing TALMaj-SssI WT or T313D (Fig 4B). We conclude that targeted DNA methylation to major satellites did not interfere with kinetochore function during early embryonic cleavage stages.

### Introduction of pericentromere DNA methylation with CRISPR/Cas9 system

**Fig 5:**
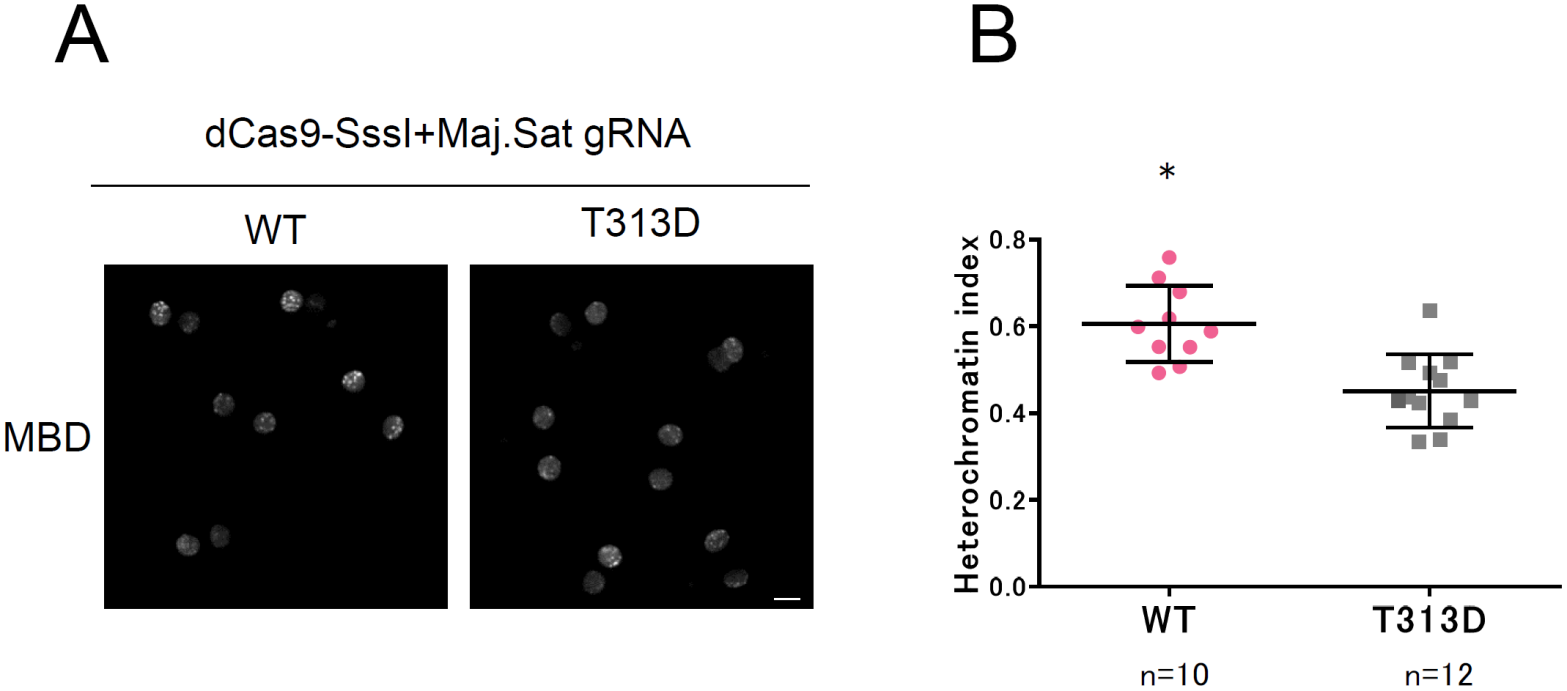
Target dominant upregulation of DNA methylation by dCas9-SssI expression in mouse embryos. (A) Live-cell imaging of DNA methylation in dCas9-SssI expressing 2-cell embryos. Fertilized embryos were injected with RNAs encoding dCas9-SssI, major satellite gRNA, mCherry-MBD-NLS and H2B-EGFP (not shown). Expression level was controlled by adjusting concentration of RNA encoding dCas9-SssI (60ng/μL) and gRNA (60ng/μL). MBD fluorescent images were captured about 4–5 h after 2-cell division. Enzymatically active dCas9-SssI (WT) and inactive dCas9-SssI (T313D) were evaluated. Scale bar represents 20μm. (B) Quantification of the heterochromatin index of embryos expressing dCas9-SssI and mCherry-MBD-NLS and H2B-EGFP. Fluorescent signal of mCherry-MBD-NLS was evaluated with 2-cell embryos obtained 17 h after 2-cell division. Asterisks indicate significant difference by Mann-Whitney *U* test (*p*<0.05).

Compared with the TALE technology, it is easier to design and construct targeting vectors with the CRISPR/Cas9 system. We expressed the fusion protein dCas9-SssI with gRNA recognizing major satellite sequences [20] in preimplantation embryos. In 2-cell stage embryos, MBD signals accumulating at pericentromeric heterochromatin were slightly higher in embryos expressing WT dCas9-SssI compared with the T313D and gRNA combination (Fig 5A). The heterochromatin index during the 2-cell stage was upregulated in the WT dCas9-SssI expressing embryos, but not in T313D expressing embryos (Fig 5B).

**Fig 6:**
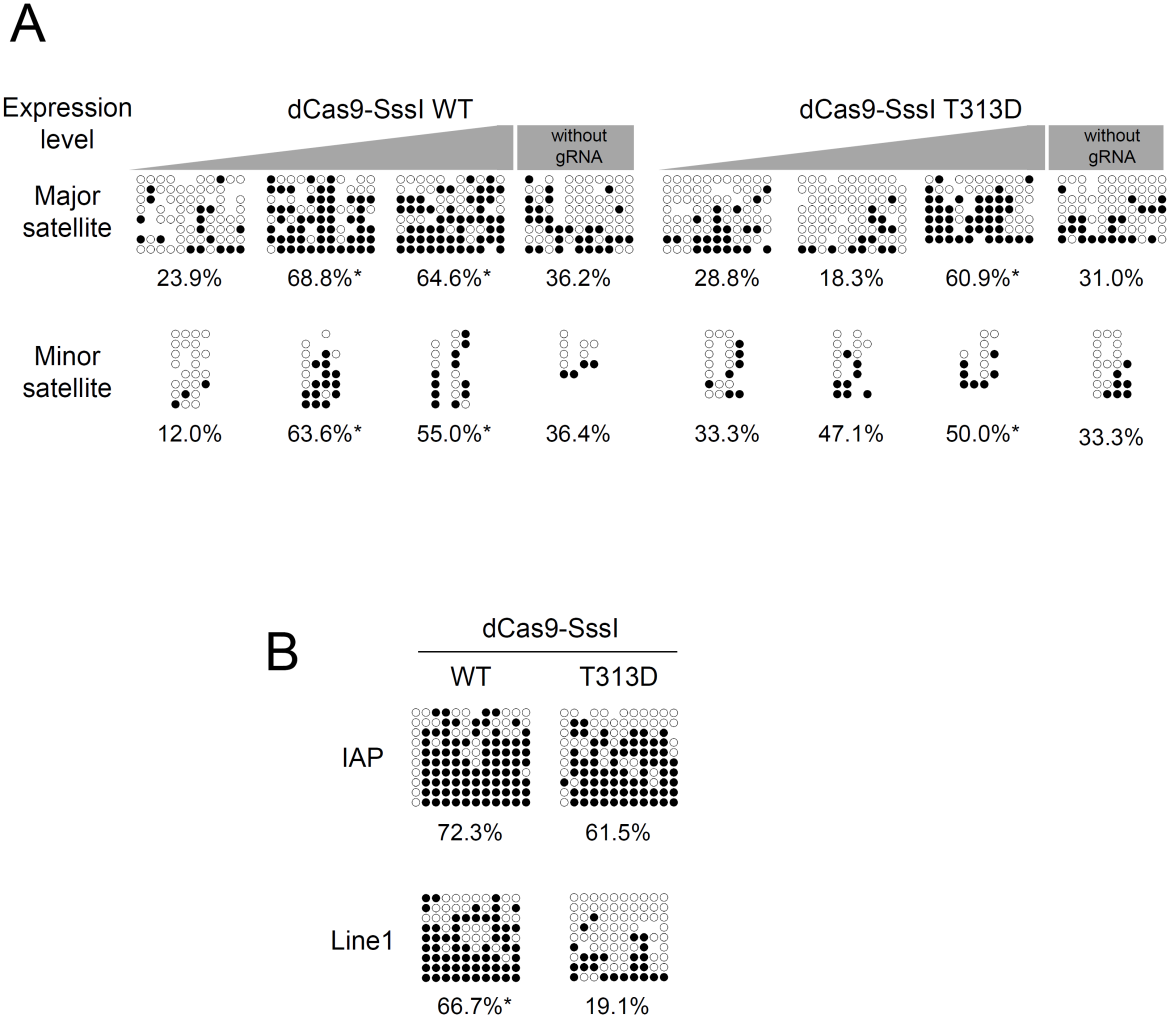
Target dominant upregulation of DNA methylation by dCas9-SssI expression with major satellite gRNA in mouse embryos. (A) Bisulfite sequence of major satellite and minor satellite CpGs of dCas9-SssI expressing and major satellite gRNA introduced embryos. Low, mid and high expression of dCas9-SssI was achieved by injection of various concentration (12ng, 60ng and 300ng/μL) of RNA. Embryos were recovered 3 days after RNA injection. In high expression condition, Embryos expressing dCas9-SssI without gRNA were also evaluated. Asterisk indicates significant difference between low and other test groups by Mann-Whitney *U* test. (B) Bisulfite sequence of IAP and Line1 CpGs of dCas9-SssI expressing and major satellite gRNA introduced embryos. Mid-level expression of dCas9-SssI was achieved by injection of 60ng/μL of RNA. Data are represented as % of methylated CpG sites per total CpG sites. Asterisk indicates significant difference between WT and T313D by Mann-Whitney *U* test.

Finally, we evaluated the specificity of DNA methylation with bisulfite sequencing (Fig 6). Although unexpected DNA methylation was also observed in embryos expressing a high dose of the T313D mutant, dose and gRNA dependent upregulation of major satellite DNA methylation was demonstrated by WT dCas9-SssI expression (Fig 6A). Line1 DNA methylation was higher than that of TALMaj-SssI expressed embryos (Figs 2B and 6B). As these results demonstrate, fewer off-target effects were shown with the TALE-based system compared with CRISPR/Cas9 when SssI methyltransferase was used as the effector domain of epigenome editing enzymes.

## Discussion

We demonstrated that it is possible to use the TALE or CRISPR/Cas9 systems to induce DNA methylation in mouse preimplantation embryos with the bacterial CpG methyltransferase, SssI. Successful induction of DNA methylation was performed in major satellite repeats located in mouse pericentromeres with the epigenome editing enzymes TALMaj-SssI or dCas9-SssI. Recently, there have been reports of inducing DNA methylation by TALE-Dnmt3a or dCas9-Dnmt3a in single copy sequences such as *Snrpn*, *p16*, *IL6ST*, or *BACH2* promoters [22, 25, 26]. This is the first report where DNA methylation has been induced in tandem copy sequence, and the upregulated DNA methylation was detectable at the fluorescent microscopic level with a mCherry-MBD-NLS probe. It is known that Dnmt3L stimulates the catalytic activity of Dnmt3a [27, 28], and it is possible that the methylation activity of Dnmt3a is dependent on the amount of Dnmt3L in host cells. By contrast, SssI does not require coupling factor to induce DNA methylation. This is the advantage of using SssI as a methylation effector in various types of cells including DNMT3L downregulated cells such as teratoma or some of the embryonal carcinoma-derived cell lines [41, 42].

In this study, embryos expressing dCas9-SssI with gRNA induced DNA methylation in major satellites, target DNA methylation was reduced when dCas9-SssI was introduced in embryos without gRNA (Fig 6A). This suggests that gRNA has an important function in tethering (d) Cas9 (fusion) proteins to the desired genomic region. This gRNA dependent DNA methylation was also observed in the dCas9-Dnmt3a system [25, 26].

Reducing the off-target effects of (epi) genome editing technology to increase the quality of experiments is a subject often discussed. With the dCas9-SssI and gRNA combination, off-target DNA methylation was induced in Line1 sequences when compared with TALE-SssI (Figs 2 and 6). These results indicate that TALE-SssI has the advantage of fewer off-target effects compared with dCas9-SssI. Optimizing the gRNA design, expression ratio of dCas9-SssI and gRNA, and moreover, applying split enzyme methods [43] and optimization of SssI amino acid residues [44], may reduce off-target effects, and these are required in our CRISPR/Cas9 system. In contrast to our study, Liu et al. reported that the CRISPR-based system has advantages over the TALE-based system in terms of editing DNA methylation [26]. They used Dnmt3a to induce DNA methylation, whereas we applied SssI as the effector. This difference between studies suggests that the efficiency of editing the epigenome using a TALE- or CRISPR-based system depends of the characteristics of fused enzyme, and that it is important to assess the target specificity of the designed enzyme. By confirming the target specificity of these enzymes, whole-genome bisulfite sequence (WGBS) is one of the best ways to assess the target specificity of DNA methylation clearly and quantitatively [45]. TALMaj-SssI bound the major satellite and Line1 retrotransposon in our ChIP-qPCR analysis (Fig 1E). This result was unexpected because TALMaj-Ty1 specifically bound the major satellite sequence in a previous ChIP-qPCR analysis [30]. We consider a possibility to explain this off-target binding of TALMaj-SssI. High expression level may induce off-target localization of TALMaj-SssI in chromatin. We used the Ef1α promoter to drive the TALMaj-SssI gene stably in Dnmt TKO ES cells. This may have been sufficient for the expression of the TALMaj-SssI gene, but too strong to drive TALMaj-SssI in cultured cells. Therefore, we evaluated DNA methylation in embryos expressing TALMaj-SssI and dCas9-SssI at various levels (Figs 2 and 6). Bisulfite sequence analysis indicated that DNA methylation was upregulated in the major satellite in TALMaj-SssI-expressing embryos, whereas Line1 DNA methylation was not when it was expressed with moderate level (10ng/μL). This result suggests that optimization of the expression level of the epigenome editing enzyme is important for reducing the off-target effect in cells. Chromosome segregation analysis also indicated that there were no significant differences in ACS frequency between the WT and T313D forms of TALMaj-SssI-expressing embryos (Fig 4). This implies that DNA methylation in pericentromeres has few effects on kinetochore or chromosome segregation during preimplantation development. Because ACS occurs in 54.1% of IVF embryos [38], the frequencies of ACS seen here (38% in the WT and 25% in the T313D forms) appears to be normal. These data also suggest that little if any leakage to minor satellites occurs when appropriate amounts of the enzyme are expressed.

In this study, the heterochromatin index was upregulated in TALMaj-SssI expressing embryos (Fig 3). This suggests that DNA methylation in pericentromeres affects heterochromatin formation in the 2-cell embryonic stage. Because loss of DNA methylation brings a slight reduction of H3K9me3 and H4K20me3 and increment of H3K27me3 in major satellites [46], it is possible that induction of DNA methylation in major satellites might lead to some repressive histone modifications such as H3K9me3 and H4K20me3 and affect chromatin configuration. There are several reports that centromeres or pericentromeres have roles in biological functions such as forming functional kinetochores [47] and genome stability in cancerous cells [48–51]. In gametogenesis, global hypomethylation, including imprinting control regions, centromeres, and pericentromeres, is observed in 13.5 dpc PGCs, whereas 10.5 dpc PGCs reveal hypermethylation in these regions [10, 52]. This demethylation, which is ensured by both passive DNA demethylation [52, 53] and replication independent DNA demethylation [54], has roles in resetting somatic memory such as genomic imprinting and establishing germ-cell specific epigenomes during gametogenesis. We focused on DNA methylation in centromeres and pericentromeres. Currently, few studies have investigated these functions because of difficulties in manipulating locus-specific DNA methylation. Our study may lead to advances in ensuring locus-specific induction of DNA methylation and may reveal the consequence and biological function of the DNA methylation status in pericentromeres in future studies.

## Supporting information

S1 Movie Live-cell imaging of DNA methylation in TALMaj-SssI expressing embryos during 1-cell to 2-cell stages. Movies of mCherry-MBD-NLS (Red; upper panel), H2B-EGFP (Green; middle panel) were indicated. Fluorescent images were captured with embryos expressing enzymatically active TALMaj-SssI (WT; left panel) or inactive TALMaj-SssI (T313D; right panel).
(MP4)

S2 Movie 3D view of mitotic chromosome of TAL-SssI expressing embryo in first mitotic division. Movies of mCherry-MBD-NLS (Red; upper panel), H2B-EGFP (Green; middle panel) were indicated. Fluorescent images were captured with embryos expressing enzymatically active TALMaj-SssI (WT; left panel) or inactive TALMaj-SssI (T313D; right panel).
(MP4)
